# Using Facebook for Improving the Psychological Well-Being of Individuals Experiencing Homelessness: Experimental and Longitudinal Study

**DOI:** 10.2196/mental.9814

**Published:** 2018-10-10

**Authors:** Fran Calvo, Xavier Carbonell

**Affiliations:** 1 Facultat de Psicologia, Ciències de l'Educació i de l'Esport Blanquerna Universitat Ramon Llull Barcelona Spain; 2 Facultat d’Educació i Psicologia Unversitat de Girona Girona Spain

**Keywords:** homelessness, individuals experiencing homelessness, health, satisfaction with life, self-esteem, self-efficacy, social networking sites, social skills

## Abstract

**Background:**

Web-based social networks are a powerful communicative element and their use is increasingly widespread. Persons living in extreme social exclusion such as individuals experiencing homelessness can benefit from the positive elements of communication and relationship associated with social networking sites.

**Objective:**

This study aimed to suggest the comparison of a Facebook training course and an office software course and their effect on psychological well-being in a group of individuals experiencing homelessness.

**Methods:**

An experimental and longitudinal study was designed. Individuals experiencing homelessness were randomly assigned to either the Facebook group or the office software group, and their social skills, self-esteem, self-efficacy, and satisfaction with life were measured on 4 occasions: pretest, at the end of the training course, 1 month later, and 3 months later. A mixed analysis of variance of repeated measures (2×4) was performed.

**Results:**

A total of 92 individuals experiencing homelessness participated in the study. The number of cases in which the 4 measurements were completed was 71 (35 in the intervention group and 36 in the control group). The mixed analysis of variance of repeated measures and the multiple regression analysis indicated a significant increase of the 4 analyzed parameters, with greater significance in the areas of social skills and self-esteem. The critical levels associated to the interaction Time×Program were significant in all variables and levels. Therefore, the scores in the 4 analyzed constructs were not equal according to the program carried out throughout the work. The effect size associated to the interaction Time×Program in the social skills scores was large (η^2^=0.32); in the self-esteem and self-efficacy scores, it was medium, (η^2^=0.13); and in the satisfaction with life scores, it was small (η^2^=0.09). The results of the adjustment of the different models of multiple linear regression indicate that the number of hours devoted weekly to the use of Facebook was a predictor of the increase in the scores of social skills (B=3.43, r^2^=.405) and self-esteem (B=.382). Age (B=.175) and self-efficacy (B=.09) were also variables, which with independence and in equal conditions, predicted self-esteem (r^2^=.29). Finally, self-esteem (B=.69) was also a predictor variable of the increase of satisfaction with life (r^2^=.195).

**Conclusions:**

These findings suggest that Facebook could be a key element in homeless psychological well-being and socialization.

## Introduction

Homelessness is a situation of extreme social exclusion with very serious organic, psychological, and social consequences. Individuals experiencing homelessness display, when compared against the general population, higher levels and more acute episodes of mental health disorders [1] and associated mortality, and their symptomatology is more severe [2]. The prevalence of substance use disorders is also higher, including the consumption of drugs parenterally, which is associated with a higher contagion of infectious diseases such as HIV, hepatitis C, or tuberculosis [3]. Individuals experiencing homelessness receive health services less frequently and display an insufficient retention in treatment, which not only worsens their evolution and prognosis [4] but also indicates that they have an especially high demand for emergency room attention, which has an effect on public health expenses [5]. Childhood traumas and family conflicts are among the main causes of homelessness [6,7]. Apart from the mentioned issues, there is an increase in the possibilities of facing marginalization, unsatisfactory personal relationships, and few possibilities for personal development.

Works on loneliness in individuals experiencing homelessness have reported high levels of interpersonal isolation and self-alienation as a consequence of a situation of constant struggle for daily survival, violence, victimization, abuse, drug consumption, and social stigma [8,9]. All this has contributed to the generalized perception that individuals experiencing homelessness are lonely people isolated from the rest of society [10]. However, in the last 10 years, there have been substantial changes in humans’ communicative and relational dimensions. Internet use has reached practically all areas of society, and access to social networking sites (SNS) has multiplied in the last 15 years. The best example of this is the dramatic rise of Facebook, the SNS with the highest number of users, which went from having 500 million users in 2010 to having 2 billion users with a registered profile in May 2017 [11].

With the incorporation of SNS in daily life, scientific investigation of their influence on human behavior has proliferated, and behaviors related to homelessness are no exception. Until mid-2012, between 44% and 62% of individuals experiencing homelessness owned a mobile phone, between 24% and 40% owned a personal computer, and between 47% and 55% used the computer and accessed the internet [12], and these numbers seem to be increasing. The prevalence of use of SNS among individuals experiencing homelessness is not too distant from that of the general population; the main motivations for their use are the access to useful information and communication with friends and family [13].

The use of SNS presents great opportunities to improve the health of individuals experiencing homelessness. Work carried out with homeless youths to prevent sexually transmitted diseases showed that there exists a high association between Web-based communication and a significant decrease in risk behavior related to HIV and hepatitis C exposure, as well as a higher perception of the risk of people exposed to the possibility of contagion of HIV and hepatitis C [14,15]. SNS are effective tools to promote the increase of individuals experiencing homelessness’ participation in the processes of community intervention [16] and are proving to be a fundamental tool in the social inclusion and prevention of homelessness among refugees [17]. Regarding mental health, promoting communication with families and friends through SNS reduces the appearance of symptoms of severe mental disorders [18], and it is an opportunity to improve psychological well-being and reduce the probabilities of suffering certain symptoms of mental illness in adults as well [19,20].

Until now, studies carried out on the use of information and communication technologies (ICT) by homeless people recommended the development of formative proposals on learning how to use them. ICT use has been linked to an increase in levels of self-esteem and self-efficacy [21], which have an effect on all areas of the person. With regards to self-esteem, first, it must be considered that self-esteem is a psychological construct of great clinical importance due to its connection with psychopathology, stress, depression, anxiety, and general well-being [22]. Self-esteem is one of the key protective factors of other serious issues related to individuals experiencing homelessness such as loneliness, risk of suicide, or other incapacitating issues [23]. Investigation has proved that the longer a person spends experiencing homelessness, the more isolated they become from social support, which creates or worsens the problems associated with homelessness [24]. Self-esteem increases when individuals experiencing homelessness overcome new challenges and have healthy relationships. The deliberate use of SNS contributes to optimizing the resocialization process, increases subjective well-being, and softens the effect of the struggles faced on the street [25]. Recommendations suggest the capacitating of individuals experiencing homelessness to improve their competences in the use of SNS so that they can benefit proactively from the protective effects associated with internet-based communication [26].

The aim of this study was to confirm whether a group of training sessions to capacitate individuals experiencing homelessness to use Facebook indicates an increase of their communicational uses and whether this improves the levels of social skills in the internet-based surrounding, self-esteem, self-efficacy, and satisfaction with life. It is expected that participants will achieve better marks in these psychological constructs and that this improvement will linger on in time.

## Methods

### Design

The study used a longitudinal prospective and experimental design, and a randomized controlled trial pretest-posttest with an intervention group and control group.

### Participants

A total of 92 individuals experiencing homelessness from a city in the northwest of Spain with approximately 100,000 inhabitants participated in the study. All participants were aged ≥18 years, and the recruitment took place from January to March 2017. The European Typology of Homelessness and Housing Exclusion (ETHOS) classification criterion was used to determine the condition of individuals experiencing homelessness [27]. The ETHOS classification is described by the European Federation of National Organizations Working with the Homeless. It includes roofless and homeless people, as well as people in inadequate or unsafe housing, illegal housing, temporarily occupied housing, or substandard housing. The sample was formed using a probability sampling method on cases that met the inclusion criteria.

### Procedure

The professionals in the harm reduction service in Girona who perform community tasks in outreach detected the cases of individuals experiencing homelessness. This team is part of the mental health and addictions public network that belongs to the Catalan government. Among others, one of its functions is to work with individuals experiencing homelessness on the street and to collaborate in shelters and at the city drop-in center, which is also public. The recruitment was carried out on the streets, in illegally occupied houses and the municipal shelter, bearing in mind the expertise that the team has in this kind of action.

The criteria of inclusion were (1) being an adult; (2) not having a Facebook account or in the event of having one, claiming to have low knowledge of its use; (3) wishing to be trained in order to improve one’s skills; (4) partaking voluntarily in the study; (5) having good Spanish language skills; and (6) being included in one of the categories of homelessness according to the ETHOS classification of homeless people.

The participants who met the criteria were assigned in a random, proportional, and stratified way, according to gender, origin (indigenous or foreign), and the weekly number of hours of use of SNS at intervention group or control group. The strata were defined bearing in mind that these are the principal variables that could bias the results of the study in the Spanish context [28]. Initially, 48 individuals experiencing homelessness were assigned to the intervention group and 44 to the control group.

For the intervention group, an educational training course aimed at learning or improving the use of Facebook was designed: creation of an email account or recovering forgotten passwords; signing up on Facebook; designing a user profile; visualizing it (how we are presented on the net); privacy options; searching for people; requesting and accepting friends; sending pictures, videos, or private messages; creating and following groups.

For the control group, a basic office software course similar to those offered by social services or nongovernmental organizations in the city to people with very serious risk of social exclusion was developed. For this purpose the contents of the 4 courses taught in different institutions in the area were gathered and homogenized: identifying the basic parts of a computer, switching it on and shutting it down, knowledge of the desktop and files, main uses of a text processer, saving documents, basic design of a curriculum vitae, access to the internet, exploring the main job search websites in Spain, creating email accounts, recovering passwords, and many more. Job search is a relevant component in office software courses offered to people at risk of residential exclusion, as work reinsertion is normally one of the main aims in the social services’ working plans.

Both courses were carried out in sessions of 1.5 hours, once a week, for 8 weeks, in 2 rooms in 2 community sociocultural centers in the city. The training sessions were developed with prospective didactics, taking into account, as much as possible, the users’ previous experience, with individualized open attention. The training experience was based on the Zone of Proximal Development concept, that is, considering *the distance between the actual developmental level as determined by independent problem solving and the level of potential development as determined through problem solving under adult guidance, or in collaboration with more capable peers* [29]. In other words, despite the fact that the contents were predesigned, the trainers were especially sensitive and flexible to the demands and needs of the participants.

In order to facilitate this methodology, the participants were divided into small groups, and 12 university students collaborated in the project (6 for intervention group and 6 for control group), acting as trainers after they completed a 20-hour training session on office software, SNS, Facebook, and homelessness. In order not to condition trainers and to avoid bias in the study, they were not informed explicitly that it was an experimental intervention group or control group design. If necessary, the students introduced cross-study contents related to the social skills involved in the topics covered: for instance, the best way to address an employer (formal email format) or examples of how to be assertive when commenting on a Facebook post.

All participants were given an informative handout on the aims of the study and signed it. The students signed a confidentiality clause so as to preserve information regarding the participants.

The pretest was carried out on the first day of training in both groups, and the first posttest on the last day, at the end. The posttests were carried out 1 month and 3 months, respectively, after the training ended. They were distributed in the shelter where the individuals experiencing homelessness received assistance, food, or sleep accommodations, or at the drop-in center where leisure and reinsertion activities were available. To assist those who were not found in these places, and to ensure maximum participation in the posttest, different professional teams of the net of attention to individuals experiencing homelessness sought the participants and reminded them systematically about the importance of the tests. In certain cases, the open medium team attended the places in which the individuals experiencing homelessness lived to ensure that the tests were completed. The investigators who administered the posttests did not participate directly in any of the training sessions in order to reduce the social desirability effect in the assessment of the participants. The investigation protocol was approved by the Ethical Committee on Biomedical Research of Girona (Cod. XSO_2017_23/05/2017).

### Instruments

The participants’ sociodemographic information and self-reports of internet use were gathered through an adaptation of a questionnaire on the use of SNS in adult populations [30]. At the end of the intervention, the participants were asked about their general satisfaction and the utility of the activity in their daily life, in 2 Likert-type questions with answer choices ranging from 1-10 (1 “unsatisfied” to 10 “very satisfied”), and whether they would partake in a second treatment to refresh the knowledge they had learned or to learn more in depth. The dependent variables were analyzed with the following instruments:

1. The Multidimensional Scale of Social Expression-C [31] was used to assess social skills in the internet context. This is a Likert-type scale of 40 items with scores from 0-4 (lower and higher frequency of the occurrence of each item), in which a higher score indicates more adaptive social skills in the internet context, for example, “I’m afraid of speaking in public and doing it badly.”
2. Self-Esteem Scale [32] is valid and reliable for Spanish populations [33] as well as for both genders and different ethnic groups [34]. It is a scale of 10 items aiming to measure global self-esteem by assessing positive and negative feelings toward oneself. All items are answered using a Likert-type scale with 4 answer choices concerning how much the participant agrees or disagrees with statements such as “I have a positive attitude toward myself.”
3. The General Self-Efficacy Scale [35] was adapted to Spanish populations [36]. It is a Likert-type scale of 10 items with answer choices from 10-100 aiming to measure the degree of general self-efficacy, defined as the feeling of social competence in effectively dealing when facing different stressful situations, for example, “I can solve difficult problems if I try hard enough.”
4. The Life Satisfaction Scale [37] was adapted and validated for Spanish populations [38]. It is a Likert-type scale of 5 items with 5 answer choices (1-5) representing the participant’s level of agreement or disagreement. It aims to assess the cognitive perception of subjective well-being through a global assessment that the participant does concerning their own life using statements such as “The circumstances of my life are very good.”

### Statistical Analysis

Central tendency and dispersion measures for the description of data were used. The sociodemographic variables for intervention group and control group were compared with the results of a chi-square test for the qualitative variables and the Student *t* test for independent and paired samples for quantitative variables. To determine the variability of measures in the scores of dependent variables social skills, self-esteem, self-efficacy, and satisfaction with life according to the type of program, a mixed analysis of variance of repeated measures (2×4) was applied for each. Observations were carried out pretest-posttest at the beginning of the training (T_1_), at the end of it (T_2_), 1 month later (T_3_), and 3 months later (T_4_). Subsequent post hoc contrasts (Bonferroni) were carried out, and the extent of the effect and potency were observed. On the other hand, the scores from each observation were correlated with the Pearson test, and a multiple linear regression model was adjusted for each of the variable dependents social skills, self-esteem, self-efficacy, and satisfaction with life including the quantitative variables age and number of hours using SNS in the model, with the aim of determining the predictor variables of increase in the analyzed psychological constructs.

## Results

### Participant Characteristics

A total of 21 participants were discarded on the grounds of not completing the posttest measures. The number of cases in which the 4 observations were completed was 71 (35 in the intervention group and 36 in the control group). Among the participants, 79% (56/71) were men, with an age average of 39 (SD 8.86) years and 55% (39/71) were foreign (not born in Spain). In the pretest, 69% (49/71) of participants claimed to have an email account and 68% (48/71) claimed to use Facebook at any time. The people with a registered profile accessed Facebook an average of 1.2 (SD 1.3) hours a week with the principal intention of contacting family (29/71, 41%) or friends (14/71, 20%) or having free time and leisure (5/71, 7%). As can be observed in Table 1, initially there were no differences regarding gender, origin, age, having an email account or not, number of hours of use of Facebook every week, and principal aim of the connection.

The repeated-measures mixed analysis of variance indicate that the critical level associated with the Time factor was <.05 in the evolution of the 4 levels of analysis of social skills, self-esteem, and satisfaction with life, but not self-efficacy. Regarding the Program factor and the interaction Time×Program, the results were significant for all variables and levels. Therefore, the scores in the 4 analyzed constructs were not equal according to the program carried out throughout the work. In the social skills scores, the effect size associated with the interaction Time×Program was large (η^2^=0.32), whereas in the self-esteem and self-efficacy scores, it was medium, (η^2^=0.13), and in the satisfaction with life scores, it was small (η^2^=0.09), following the Lacobucci interpretation [39]. The potency observed was very high in all cases (social skills=1, self-esteem=1, self-efficacy=1, satisfaction with life=.98). These results can be observed in Table 2.

### Social Skills

**Figure 1:**
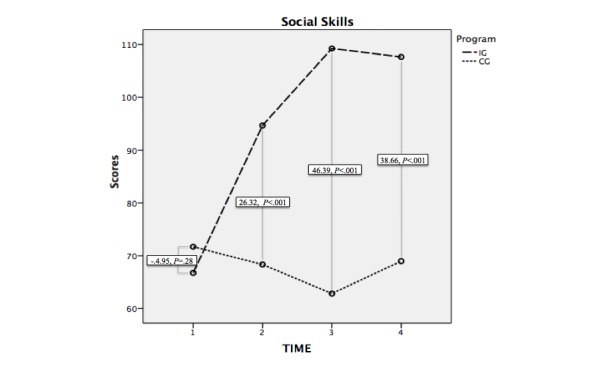
Evolution of the scores in social skills in the intervention and control groups and the difference of averages at each level of analysis. IG: intervention group; CG: control group.

The difference in averages in the Program factor (intervention group−control group) was 26.61 points (*F*_1,69_=33.04, *P*<.001). The post hoc analysis (Bonferroni) indicated that in the Time×Program interaction, albeit there were no significant differences between the scores of both groups when the pretest was carried out (*F*_1,69_=.76, *P*=.39), differences did exist at the end of the intervention (*F*_1,69_=26.58, *P*<.001), 1 month later (*F*_1,69_=57.19, *P*<.001), and 3 months later (*F*_1,69_=41.04, *P*<.001). The difference in averages at each level may be observed in the graphic representation of the evolution of scores (Figure 1).

**Table 1:** Descriptive data of the sample and comparison of the intervention group and the control group by sociodemographic variables and pretest use of Facebook.

Sociodemographic variables and pretest Facebook use		Intervention group (n=35)	Control group (n=36)	Total sample (n=71)	Intervention group and control group comparison		
					χ^2^or *t*	df	*P* value
**Sex, n (%)**					0.66	1	.30
	Male	29 (41)	27 (38)	56 (79)	—^a^	—	—
	Female	6 (8)	9 (13)	15 (21)	—	—	—
**Origin, n (%)**					0.34	1	.37
	Indigenous	17 (24)	15 (21)	32 (45)	—	—	—
	Foreign	18 (25)	21 (30)	39 (55)	—	—	—
Registered email, n (%)		24 (34)	25 (35)	49 (69)	0.006	1	.57
Age, mean (SD)		38.94 (9.11)	39.14 (8.74)	39.04 (8.86)	−0.093	69	.93
Hours per week using Facebook, mean (SD)		1.20 (1.35)	1.19 (1.31)	1.20 (1.32)	0.018	69	.99
**Use of Facebook, n (%)**		26 (37)	22 (31)	48 (68)	1.43	1	.58
	To contact family	14 (20)	15 (21)	29 (41)	0.84	1	.42
	To contact friends	9 (13)	5 (7)	14 (20)	0.73	1	.39
	For leisure	3 (4)	2 (3)	5 (7)	1.23	1	.51

^a^Not applicable.

**Table 2:** Descriptive statistics and results of mixed repeated measurements analysis of variance.

Analyzed constructs		Intervention, mean (SD)	Control, mean (SD)	Phase comparison, *F*_1,69_					
				Time		Program		Time×Program	
				*F* (η^2^/Op^a^)	*P* value	*F* (η^2^/Op)	*P* value	*F* (η^2^/Op)	*P* value
**Social skills**				18.61 (0.21/1)	<.001	33.14 (0.32/1)	<.001	32.81 (0.32/1)	<.001
	T_1_^b^	66.74 (4.05)	71.69 (3.99)	—^c^	—	—	—	—	—
	T_2_^d^	94.66 (3.64)	68.33 (3.58)	—	—	—	—	—	—
	T_3_^e^	109.23 (4.37)	62.83 (4.31)	—	—	—	—	—	—
	T_4_^f^	107.63 (4.30)	69.97 (4.24)	—	—	—	—	—	—
**Self-esteem**				4.22 (0.03/.31)	.006	10.15 (0.10/.77)	<.001	7.47 (0.13/1)	<.001
	T_1_	20.37 (0.87)	21.86 (0.86)	—	—	—	—	—	—
	T_2_	24.49 (0.97)	19.91 (0.95)	—	—	—	—	—	—
	T_3_	23.11 (0.90)	17.83 (0.88)	—	—	—	—	—	—
	T_4_	20.94 (0.87)	18.86 (0.86)	—	—	—	—	—	—
**Self-efficacy**				2.28 (0.03/.57)	.08	26.26 (0.98/.99)	<.001	10.03 (0.13/1)	<.001
	T_1_	53.03 (1.96)	55.36 (1.93)	—	—	—	—	—	—
	T_2_	63.69 (2.00)	51.61 (1.98)	—	—	—	—	—	—
	T_3_	65.80 (1.80)	50.44 (1.78)	—	—	—	—	—	—
	T_4_	63.43 (2.10)	52.39 (2.07)	—	—	—	—	—	—
**Satisfaction with life**				5.96 (0.08/.95)	.001	7.01 (0.09/.75)	<.001	7.23 (0.09/.98)	<.001
	T_1_	10.63 (0.56)	11.14 (0.55)	—	—	—	—	—	—
	T_2_	13.86 (0.65)	11.28 (0.64)	—	—	—	—	—	—
	T_3_	14.31 (0.58)	10.67 (0.57)	—	—	—	—	—	—
	T_4_	13.99 (0.61)	12.83 (0.60)	—	—	—	—	—	—

^a^Observed potency.

^b^T_1_: Observations performed pretest-posttest at the beginning of the training.

^c^Not applicable.

^d^T_2_: Observations performed at the end of training.

^e^T_3_: Observations performed 1 month later.

^f^T_4_: Observations performed 3 months later.

The effect on the intragroup tests was significant in the measures T_1_-T_2_(*F*_1,69_=28.96, *P*<.001), T_2_-T_3_(*F*_1,69_=16.85, *P*<.001), T_2_-T_4_(*F*_1,69_=4.48, *P*=.038), T_1_-T_3_(*F*_1,69_=67.65, *P*<.001), and T_1_-T_4_(*F*_1,69_=47.01, *P*<.001). The measure between levels T_3_-T_4_ displayed a tendency toward significance (*F*_1,69_=3.78, *P*=.06).

The analysis of averages for related tests for each group showed that in the intervention group, the average difference between T_1_-T_2_ was −27.91 points (SD 28.97, *t*_34_=−5.70, *P*<.001), between T_2_-T_3_ was −14.57 (SD 20.91, *t*_34_=−4.12, *P*<.001), between T_1_-T_3_ was −42.49 (SD 28.55, *t*_34_=−8.80, *P*<.001), and between T_1_-T_4_ was −40.89 (SD 27.13, *t*_34_=−8.92, *P*<.001). Table 3 displays the Student *t* test analysis for related samples carried out for each of the dependent variables. We can say that the program was effective at increasing social skills for the intervention group but not the control group, and this improvement lingered on in time until 3 months after the intervention.

### Self-Esteem

In the case of self-esteem, the difference between the averages in the Program factor (intervention group−control group) was 2.61 points (*F*_1,69_=7.47, *P*=.008). In the pretest, there were no differences in the interaction Time×Program in the scores of the 2 groups (F_1,69_=.76, *P*=.39). In the observation T_2_ corresponding with the posttest, there were differences between the averages (*F*_1,69_=11.34, *P*=.001) and likewise in T_3_(*F*_1,69_=17.62, *P*<.001). In T_4_, despite a slight tendency toward significance, there were no observed differences (*F*_1,69_=2.91, *P*=.092).

In the intragroup tests, there were significant differences in the observations corresponding to T_1_- T_2_(*F*_1,69_=17.02, *P*<.001), T_2_-T_4_(*F*_1,69_=4.66, *P*=.02), and T_1_-T_3_(*F*_1,69_=25.47, *P*<.001) and also T_1_-T_4_(*F*_1,69_=16.26, *P*<.001) and T_3_-T_4_(*F*_1,69_=4.62, *P*=.02). There was no significance in the level T_2_-T_3_(F_1,69_=.39, *P*=.54).

**Figure 2:**
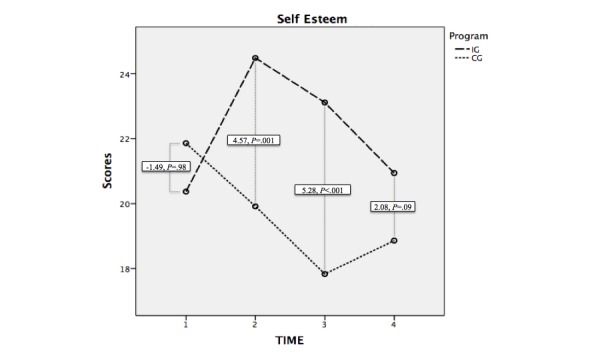
Evolution of the scores in self-esteem in the intervention and control groups and the difference of averages at each level of analysis. IG: intervention group; CG: control group.

Although the Student *t* test for paired samples analysis indicated an increase of average scores in the intervention group of −2.74 between T_1_-T_3_(SD 5.85, *t*_34_=-2.77, *P*=.01), it did not happen with T_1_-T_4_: mean −0.57 (SD 3.90), *t*_34_=−87, *P*=.39. Therefore, the effect of the intervention expired between 1 and 3 months after the intervention. On the other hand, a decrease in the average scores of self-esteem in the control group between T_2_-T_3_ (mean 2.05, SD 4.88, *t*_36_=2.56, *P*=.02), T_1_-T_3_ (mean 3.86, SD 5.46, *t*_36_=4.30, *P*<.001), and T_1_-T_4_ (mean 2.89, SD 3.57, *t*_36_=4.92, *P*<.001) was observed (Figure 2).

### Self-Efficacy

Regarding self-efficacy, there was a difference in the averages in Program (intervention group−control group), of 9.03 points (*F*_1,69_=26.26, *P*<.001). No differences were observed in time regarding the averages between groups T_1_(F_1,69_=.72, *P*=.40). There were differences between T_2_(*F*_1,69_=18.41, *P*<.001), T_3_(*F*_1,69_=36.73, *P*<.001), and T_4_(*F*_1,69_=13.99, *P*<.001).

**Figure 3:**
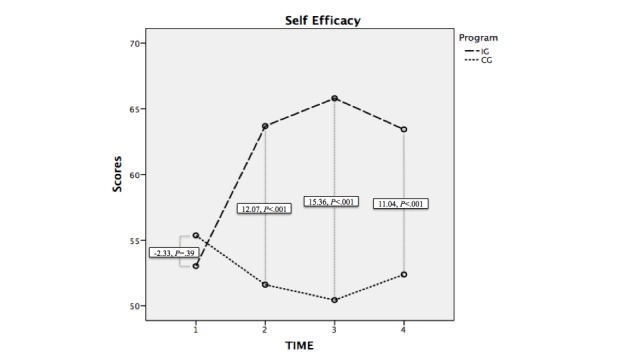
Evolution of the scores in self-efficacy in the intervention and control groups and the difference of averages at each level of analysis. IG: intervention group; CG: control group.

In the intragroups test, there were significant differences between T_1_- T_2_(*F*_1,69_=29.61, *P*<.001), T_1_-T_3_(*F*_1,69_=24.99, *P*<.001), and T_1_-T_4_(*F*_1,69_=13.15, *P*=.001), but not in the rest of the levels (T_2_-T_3_: *F*_1,69_=1.32, *P*=.25; T_3_-T_4_: *F*_1,69_=1.20, *P*=.28; T_2_-T_4_: F_1,69_=.07, *P*=.78); see Figure 3.

**Table 3:** Comparison of paired samples (t) of the different observations.

Analyzed constructs		Intervention group			Control group		
		Mean (SD)	*t*_34_	*P* value	Mean (SD)	*t*_36_	*P* value
**Social skills**							
	T_1_^a^-T_2_^b^	−27.91 (28.97)	−5.70	<.001	2.27 (20.00)	.690	.49
	T_2_-T_3_^c^	−14.57 (20.91)	−4.12	<.001	3.97 (22.51)	1.10	.28
	T_3_-T_4_^d^	1.6 (14.94)	.634	.53	−5.49 (18.53)	−1.80	.08
	T_1_-T_3_	−42.49 (28.55)	−8.80	<.001	6.24 (28.45)	1.33	.19
	T_1_-T_4_	−40.89 (27.13)	−8.92	<.001	.76 (28.71)	.160	.87
**Self-esteem**							
	T_1_-T_2_	−4.11 (6.37)	−3.82	.001	1.81 (5.98)	1.84	.07
	T_2_-T_3_	1.37 (4.69)	1.73	.09	2.05 (4.88)	2.56	.02
	T_3_-T_4_	2.17 (6.62)	1.94	.06	−.973 (5.83)	.317	.32
	T_1_-T_3_	−2.74 (5.85)	−2.77	.009	3.86 (5.46)	4.30	<.001
	T_1_-T_4_	−.571 (3.89)	−.867	.39	2.89 (3.57)	4.92	<.001
**Self-efficacy**							
	T_1_-T_2_	−10.66 (14.27)	−4.42	<.001	3.68 (6.81)	3.28	.002
	T_2_-T_3_	−2.11 (12.48)	−1.00	.32	1.57 (11.69)	.816	.42
	T_3_-T_4_	2.37 (16.03)	.875	.38	−2.38 (17.12)	−.845	.41
	T_1_-T_3_	−12.77 (15.56)	−4.86	<.001	5.24 (14.18)	2.25	.03
	T_1_-T_4_	−10.40 (14.41)	−4.27	<.001	2.86 (16.33)	1.07	.29
**Satisfaction with life**							
	T_1_-T_2_	−3.23 (3.50)	−5.50	<.001	−.054 (3.24)	−.101	.92
	T_2_-T_3_	−.457 (2.47)	−1.10	.28	.568 (4.21)	.821	.41
	T_3_-T_4_	1.31 (4.50)	1.73	.09	−1.08 (4.64)	−1.24	.03
	T_1_-T_3_	−3.69 (3.10)	−7.05	<.001	.510 (4.83)	.647	.52
	T_1_-T_4_	−2.37 (4.24)	−3.31	<.001	−1.57 (5.67)	−1.68	.11

^a^T_1_: Observations performed pretest-posttest at the beginning of the training.

^c^T_2_: Observations performed at the end of training.

^d^T_3_: Observations performed 1 month later.

^e^T_4_: Observations performed 3 months later.

**Figure 4:**
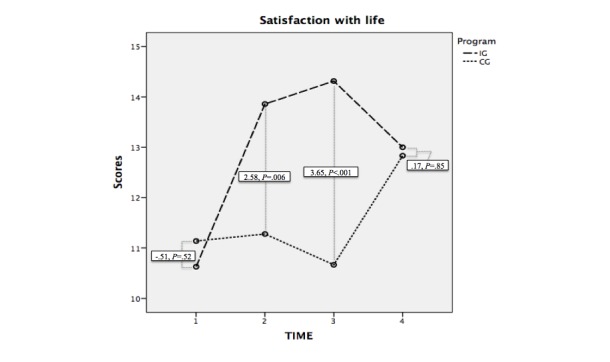
Evolution of the scores in satisfaction with life in the intervention and control groups and the difference of averages at each level of analysis. IG: intervention group; CG: control group.

The independent measures made for each of the groups displayed significant average differences in both groups in the T_1_- T_2_ comparison (intervention group: mean −10.66, SD 14.27, *t*_34_=-4.42, *P*<.001; control group: mean 3.68, SD 6.81, *t*_36_=3.28, *P*=.002). In the intervention group, the self-efficacy scores increased from the pretest to the observations 1 month later (T_1_-T_3_: mean −12.77, SD 15.56, *t*_34_=-4.86, *P*<.001) and 3 months later (T_1_-T_4_: mean −10.40, SD 14.41, *t*_34_=−4.27, *P*<.001). Contrarily, in the control group, they decreased in the measurement 1 month later (T_1_-T_3_: mean 5.24, SD 14.18, *t*_36_=2.25, *P*=.03).

### Satisfaction With Life

Regarding satisfaction with life, the Program factor displayed a difference of averages (intervention group-control group) of 1.47 points (*F*_1,69_=7.23, *P*=.009). In the post hoc tests, as can be observed in Figure 4, no intergroup average differences were found in the first level of analysis (T_1_: F_1,69_=.42, *P*=.52) or in the last level (T_4_^:^F_1,69_=.038, *P*=.85). Contrarily, differences were found in T_2_(*F*_1,69_=8.04, *P*=.006) and in T_3_(*F*_1,69_=19.88, *P*<.001).

In the intragroup tests, there were significant differences between T_1_-T_2_(*F*_1,69_=14.90, *P*<.001), T_1_-T_3_(*F*_1,69_=18.21, *P*<.001), T_2_-T_4_(*F*_1,69_=4.08, *P*=.05), and T_3_-T_4_(*F*_1,69_=8.13, *P*=.006). Contrarily, no differences were found between T_1_-T_4_(F_1,69_=.32, *P*=.57) and T_2_-T_3_(*F*_1,69_=1.66, *P*=.20).

The independent measurements carried out with the Student *t* test for paired samples in each group displayed differences in the averages in T_1_- T_2_(intervention group: mean −3.23, SD 3.50, *t*_34_=−5.50, *P*<.001), T_1_-T_3_(mean −3.69, SD 3.10, *t*_34_=−7.05, *P*<.001), and T_1_-T_4_(mean −2.37, SD 4.24, *t*_34_=−3.31, *P*=.002). No differences were found in the control group.

### Descriptive Posttest and Multiple Lineal Regression Analysis

The comparison of averages for paired samples carried out in the intervention group and control group to verify the variability in the weekly use of Facebook indicated that in both groups there was an increase in the number of hours. Between the pretest and the posttest at the end of the training course, participants in the intervention group increased their use of Facebook an average of 8 hours a week (SD 3, *t*_34_=−13.11, *P*<.001), and this number continued increasing at the end of the course, until 3 months later it reached ≥2 hours (SD 3, *t*_34_=−3.44, *P*=.002), with a total increase for T_1_-T_4_ of 10 hours a week (SD 4, *t*_34_=−13.67, *P*<.001). The control group, on the other hand, did not display an increase in hours of use from the pretest to the end of the training course (*t*_36_=0.15, *P*=.88), but it did 3 months later, with an average of 1 hour a week (SD 2, *t*_36_=−4.81, *P*<.001). The average number of hours of use 3 months after the end of the training course was significantly higher in the intervention group than in the control group (intervention group=11 hours vs control group=2 hours; *t*_69_=9.35, *P*<.001). The variables corresponding to the weekly number of hours and age were also included in the linear regression model. The correlations corresponding to each of the quantitative variables in each level of observation (pretest and 3 posttests) were carried out with the *r* for the Pearson test. These data can be seen in Multimedia Appendix 1.

**Table 4:** Multiple linear regression for the dependent variables of social skills, self-esteem, self-efficacy, and satisfaction with life.

Dependent variables of the model		Values					Collinearity	
		B	Estimated error	beta	*t_5_*	*P* value	Tolerance	Variance inflation factor
**Social skills (r^**2**^=.405)**								
	Age	.154	.369	.043	.416	.68	.870	1.149
	Use of Facebook (hrs/wk)	3.43	.599	.613	5.72	<.001	.796	1.256
	Self-esteem	−1.32	.675	–.216	−1.96	.06	.752	1.330
	Self-efficacy	.319	.248	.136	1.29	.20	.825	1.212
	Satisfaction with life	−.882	.856	−.100	−1.03	.31	.978	1.023
**Self-esteem (r^**2**^=.290)**										
	Age	.175	.062	.297	2.81	.007	.973	1.028
	Use of Facebook (hrs/wk)	.382	.122	.418	3.12	.003	.609	1.642
	Social skills	−.042	.022	−.258	−1.96	.06	.630	1.588
	Self-efficacy	.090	.043	.233	2.07	.04	.858	1.166
	Satisfaction with life	−.041	.154	−.029	−.269	.79	.963	1.038
**Self-efficacy (r^**2**^=.195)**								
	Age	−.247	.180	−.162	−1.375	.17	.893	1.120
	Use of Facebook (hrs/wk)	.371	.360	.156	1.03	.31	.538	1.858
	Social skills	.078	.061	.183	1.29	.20	.610	1.639
	Self-esteem	.688	.333	.265	2.07	.04	.757	1.321
	Satisfaction with life	.096	.426	.026	.225	.82	.963	1.039
**Satisfaction with life (r^**2**^=.038)**								
	Age	.053	.053	.131	1.01	.31	.882	1.134
	Use of Facebook (hrs/wk)	.008	.106	.012	.073	.94	.530	1.888
	Social skills	−.018	.018	−.161	−1.03	.31	.605	1.654
	Self-esteem	−.027	.100	−.039	−.269	.79	.711	1.407
	Self-efficacy	.008	.036	.031	.225	.82	.805	1.242

The results of the adjustment of the different models of multiple linear regression indicate that the number of hours devoted weekly to the use of Facebook was a predictor of the increase in the scores of social skills (B=3.43, r^2^=.405) and self-esteem (B=.382). Age (B=.175) and self-efficacy (B=.09) were also variables that, with independence and in equal conditions, predicted self-efficacy (r^2^=.29). Finally, self-esteem (B=.69) was a predictor variable of the increase of self-efficacy (r^2^=.195; Table 4).

The number of participants in the intervention group that used Facebook 3 months after the training course increased when compared with the pretest by 27%, rising from 26 to 33 people. Of the 33 people, 88% (29/33) claimed that their principal motivation for using SNS was to communicate with another person: 57% (19/33) with family and 30% (10/33) with friends. The rest used SNS for leisure or regular access to information (4/33; 12%).

Finally, the general satisfaction with the course results did not show score differences between the groups (intervention group=7, SD 1, control group=7, SD 1, *t*_69_=−.33, *P*=.74), although there did exist differences in the usefulness perceived by participants (intervention group=8, SD 1, control group=5, SD 2, *t*_69_=7.31, *P*<.001). A total of 7 participants of the intervention group expressed their refusal to repeat the training course to learn concepts more in depth, compared with 15 participants of the control group (χ^2^_1_=3.98, *P*=.05).

## Discussion

There are no known precedents of experimental longitudinal studies with homeless people in which researchers have analyzed the effects of a training program on social skills in the internet context, self-esteem, self-efficacy, and satisfaction with life. The contents of the program in this study were based on the learning or improvement of the use of Facebook, compared with a series of sessions of basic office automation and Web-based job search. For this purpose, the scores of the analyzed psychological constructs obtained in the pretest were compared with those from 3 posttest observations (end of treatment and one-month and three-month follow-ups).

The results obtained indicate that improving the use of Facebook contributes to the improvement of the psychological constructs in individuals experiencing homelessness and that this improvement does not disappear until at least 1 month after the intervention (up to 3 months in the case of social skills and self-efficacy). Therefore, the expected results are verified: participants in the program improved their scores in social skills, self-esteem, self-efficacy, and satisfaction with life, and this improvement continued over time. The improvement of the social skills in the internet context was accentuated in comparison to the rest of the analyzed variables. An analysis of the general population for any differences in social skills in the internet context and in “real” context revealed the existence of significant discrepancies in the results of both in inversely proportional relations [29]. This fact contributes to increase the mistrust that SNS generate as a risk factor with a potential to increase loneliness [40]. Nevertheless, the results obtained for individuals experiencing homelessness indicated that the people who devoted more time to Web-based social contact improved their social skills in this context, namely, the internet context, proving the effect on social skills in the real context. It must be said that even though behavior concerning the use of Facebook could be inversely related to the 2 types of social skills in the general population, we would not consider this conduct initially as maladaptive in individuals experiencing homelessness. In extremely hostile surroundings such as the ones faced on the street, communication through SNS contributes to the reduction of the tensions generated as a consequence of the discrepancies of the “real me” and the “virtual me,” as a result of the space of freedom of expression created in an environment with minimal face-to-face relationships [41].

This fact is very interesting in the analysis of the social relationships of individuals experiencing homelessness who preserve their secure and private space with mistrust. More prosocial and healthy relationships are generated in the SNS, as opposed to relationships in their closest surroundings, the street, or special centers devoted to offer social assistance [42]. Thus, the training of social skills in the internet context must be considered interrelated to relationships with real people, and not so much as limiting relationships. This is a starting point to homogenize the social abilities to develop, both in the internet context and in the “real” context, especially for individuals experiencing homelessness, who may have close relationships that are not very healthy and live in a historic period in which human development fields are taking place increasingly through the internet and SNS.

Learning to use Facebook fulfills two objectives: to improve the process of resocialization, soothing the effect of the hard situations faced, and to increase self-esteem. The reasons for this are access to information that the individual can filter and on the other hand and more importantly to provide individuals experiencing homelessness a way to contact friends and relatives through SNS [22]. Self-esteem is highly related to self-efficacy. In fact, the high tolerance indicates a high correlation of both variables but without the presence of problems associated with collinearity, which are established with values <0.1 [43]. People’s feelings and actions are affected by self-efficacy expectations. People with low levels of self-efficacy have negative feelings on their own capacities and consequently their self-esteem. The perception of self-efficacy facilitates positive thoughts of one’s capacities, motivating more challenging and persistent actions [35]. As we have seen, self-esteem and self-efficacy improve with the learning of SNS, but not with the basic office software training course, as has been suggested.

Other known proposals in basic office software training courses among individuals experiencing homelessness that explored the experience of using computers with a group of individuals experiencing homelessness without experience in the use of ICT, produced great acceptation and good attitude toward ICT [21]. After the training period, the participants claimed to have higher self-esteem and self-efficacy, although it was considered that self-efficacy was perceived in relation to a given situation (the use of technology). However, in our case, the construct has been considered in the wide sense, understanding self-efficacy as a global construct that refers to the stable belief that an individual has their own capacity to deal adequately with a series of stressors in daily life [35]. It is important to point out that the contents of the training assessed by Miller et al [21], despite displaying a component of priority search of employment, were based on occupational therapy strategies, unlike our case, in which the therapeutic factor was not considered (as is the case in the close context, in which trainings on job searching for vulnerable groups is based on the transmission of contents).

Thus, in basic office software training courses for people at risk of social exclusion, it is usually first assumed that unemployment is one of the main causes of homelessness, both in individuals experiencing homelessness and in the general population and second that the aim of the professionals who design the course is to reduce homelessness [28].

Nevertheless, despite the fact that unemployment is one of the structural causes of homelessness and access to employment increases the possibilities of inclusion in the general population, there should be an assessment of the most adequate strategies for job searching through ICT, especially with individuals experiencing homelessness and their different typologies.

Other studies support the finding that interventions based on specific occupational therapy for individuals experiencing homelessness are effective, given the distinctive therapeutic features of this type of intervention, which is not merely a training course [44]. In fact, as we have observed in the results of this study, interventions that consist exclusively of training could be counterproductive. The specific tools for Web-based job searching alone do not improve access to the work market in the general population substantially—not to mention in people with difficulties. However, in recent years, they have improved in this regard. Contrarily, contact with friends and relatives through SNS, and even job searching directly through SNS, alongside the use of specific websites and searching for work outside the internet context, results in a remarkable increase in the possibilities of employability [45], which is a factor to be taken into consideration when designing training action plans for individuals experiencing homelessness.

Regarding satisfaction with life, it is noteworthy to point out that the perception that individuals experiencing homelessness have of satisfaction with life is significantly lower than that of the general population [46]. These reduced levels of satisfaction with life are related to the housing situation, sociodemographic characteristics such as age, gender, mental and physical health, and the type of social attention received.

This complex situation is coherent with the results of this study. The levels of satisfaction with life can rise at the beginning of the program and are connected with improvement in the rest of the constructs. Satisfaction with life is the variable that is weaker longitudinally, and probably, the recovery of basic levels is related to other fields that are not directly related to communication through SNS.

In brief, SNS are presented as a channel of communication, relation, and access to information with a great potency to reinforce individuals emotionally and socially [47]. Everybody wishes to have quality relationships, and SNS help individuals experiencing homelessness to generate and maintain this type of relationship. Moreover, they have great importance in their recovery and stabilization, even in the case of people with severe mental disorders or drug addiction, whose prognosis is very often less encouraging [48].

Teaching how to use or offering a training course to improve the use of SNS, in this case, Facebook, increases exposure to this type of communication, which has the effect of increasing important indicators of psychological well-being. Contact with people who are important to individuals experiencing homelessness, such as friends and family, is of great value to them, and the contact has significant impact levels when carried out totally or partially through SNS [14]. Prosocial contact with relatives and friends through Facebook increases protective factors of certain damage associated with homelessness and its risk behaviors. On the other hand, the lack of this type of important contact increases depressive symptoms, which prompt a decrease in levels of self-esteem, self-efficacy, and even satisfaction with life [18,49].

Consequently, there is coincidence with other studies as regards the recommendation of providing more public spaces and free and quality Wi-Fi spots to facilitate access to ICT and SNS for individuals experiencing homelessness and other groups facing extreme social exclusion, on the grounds of the multiple benefits associated with internet access [25,50].

This work has some limitations. First, despite the fact that we have proved that the use of Facebook improves scores in the psychological constructs, we do not know what type of connections and what contents in communication through SNS are the most adequate to improve the well-being of participants, which could be considered a limitation to our work. The second limitation is that our sample was limited and the intervention context is specific, which makes it difficult to extrapolate the results to the group of individuals experiencing homelessness, especially owing to multiple situations, of a highly different nature, which adhere to the concept of homelessness according to the ETHOS classification. Finally, it would be helpful to extend the follow-up period to 6 months.

Future lines of research have arisen from this work. First, we consider it interesting trying to replicate this work in different contexts. Second, it would be interesting to assess the effect of job search in individuals experiencing homelessness through SNS, in comparison with specific websites, such as the ones on which our control group proposal is based. Third, the use of mixed designs in which qualitative information can be accessed in parallel with adequate quantitative models is supported, in order to complement the information obtained with the contents of the type of connections that individuals experiencing homelessness maintain.

In conclusion, training courses for the use of SNS improve social skills in the internet context, self-esteem, self-efficacy, and satisfaction with life; this improvement continues 4-12 weeks after the intervention. Furthermore, the increase in SNS use that derives from the training course becomes a predictor element of improvement of social skills and self-esteem, the latter having a positive effect on self-efficacy. The increase of these constructs is related to a decrease in individuals experiencing homelessness’ levels of loneliness, isolation, and failure, and therefore, conducting training courses for individuals experiencing homelessness to improve their experience in the use of SNS improves their quality of life and psychological state and is an interesting educational offer for institutions that provide specific services.

## Appendix

Multimedia Appendix 1 Correlation matrix (r) of dependent variables in the four steps of observation.

## Abbreviations

ETHOS: European Typology of Homelessness and Housing Exclusion
ICT: information and communication technologies
SNS: social networking sites
