# Supplementary material for: Using Facebook for Improving the Psychological Well-Being of Individuals Experiencing Homelessness: Experimental and Longitudinal Study
Source: JMIR Ment Health. 2018 Oct 10;5(4):e59. doi: 10.2196/mental.9814 (PMC6231733; doi:10.2196/mental.9814)
Supplement: Multimedia Appendix 1 [file mental_v5i4e59_app1.pdf]

Table 4.

Correlation matrix (r) of dependent variables on the four steps of observations.

| | Variables | (1) | (2) | (3) | (4) | (5) | (6) | (7) | (8) | (9) | (10) | (11) | (12) | (13) | (14) | (15) | (16) | (17) | (18) | (19) | (20) | (21) |
|---|---|---|---|---|---|---|---|---|---|---|---|---|---|---|---|---|---|---|---|---|---|---|
| | (1) Age | 1 | | | | | | | | | | | | | | | | | | | | |
| Pre test | (2) Facebook | .030 | 1 | | | | | | | | | | | | | | | | | | | |
| | (3) SS | -.145 | .031 | 1 | | | | | | | | | | | | | | | | | | |
| | (4) SE | .168 | .223 | .129 | 1 | | | | | | | | | | | | | | | | | |
| | (5) SF | .019 | -.011 | .052 | .321[b] | 1 | | | | | | | | | | | | | | | | |
| | (6) SL | -.130 | -.166 | -.071 | -.132 | -.240[a] | 1 | | | | | | | | | | | | | | | |
| Post test Finish | (7) Facebook | .022 | .267[a] | .031 | .102 | -.013 | -.090 | 1 | | | | | | | | | | | | | | |
| | (8) SS | .095 | 040 | .303[a] | -.063 | .048 | -.141 | .381[b] | 1 | | | | | | | | | | | | | |
| | (9) SE | -.037 | 068 | .047 | .273[a] | .094 | .017 | .330[b] | .283[a] | 1 | | | | | | | | | | | | |
| | (10) SF | .018 | 066 | .437[c] | .164 | .437[c] | -.047 | .459[c] | .304[a] | .318[b] | 1 | | | | | | | | | | | |
| | (11) SL | -.156 | -.404 | -.073 | -.225 | -.195 | .505[c] | 173 | -.026 | .015 | -.073 | 1 | | | | | | | | | | |
| Post test 1 month | (12) Facebook | .016 | .339[b] | -.010 | .091 | -.044 | -.056 | .928[c] | .386[c] | .370[b] | .519[c] | .114 | 1 | | | | | | | | | |
| | (13) SS | .'15 | 063 | .257[a] | -.175 | -.066 | -.178 | .500[c] | .754[c] | .263[a] | .282[a] | .124 | .529[c] | 1 | | | | | | | | |
| | (14) SE | -.120 | 031 | .005 | .303[b] | .096 | .024 | 461[c] | .258[a] | .682[c] | .384[b] | .138 | .512[c] | .319[b] | 1 | | | | | | | |
| | (15) SF | .040 | -.121 | -.120 | .029 | .025 | .008 | 564[c] | .267[a] | .235[a] | .581[c] | .152 | .597[c] | .310[b] | .400[b] | 1 | | | | | | |
| | (16) SL | -.183 | -.194 | -.106 | -.230 | -.230 | .192 | 343[b] | .140 | .013 | -.043 | .606[c] | .318[b] | .407[c] | .237[a] | .217 | 1 | | | | | |
| Post test 3 months | (17) Facebook | .033 | .432[c] | -.006 | .108 | -.024 | -.089 | .880[c] | .354[b] | .394[b] | .534[c] | .048 | .841[c] | .492[c] | .475[c] | .511[c] | .202 | 1 | | | | |
| | (18) SS | -.024 | 223 | .261[a] | -.160 | -.126 | -.159 | .523[c] | .634[c] | .226 | .363[b] | .047 | .577[c] | .872[c] | .252[b] | .323[b] | .320[b] | .593[c] | 1 | | | |
| | (19) SE | .295[a] | 160 | .107 | .686[c] | .130 | -.089 | .314[b] | .109 | .222 | .291[b] | -.021 | .335[b] | .031 | .335[b] | .154 | -.012 | .360[b] | .056 | 1 | | |
| | (20) SF | -.080 | 088 | .046 | .281[a] | .108 | -.265 | .394[c] | .322[b] | .282[a] | .225 | -.029 | .390[b] | .250[a] | .356[b] | .221 | .204 | .353[b] | .291[b] | .283[b] | 1 | |
| | (21) SL | .121 | -.174 | -.150 | .129 | .137 | -.056 | .045 | .036 | .023 | .031 | .094 | -.075 | -.086 | -.031 | .007 | -.039 | -.082 | -.150 | .004 | -.033 | 1 |

[a]p < .05, [b]p < .01, [c]p < .001
